# Incidence of Diabetic Nephropathy and Its Predictors among Type 2 Diabetes Mellitus Patients at University of Gondar Comprehensive Specialized Hospital, Northwest Ethiopia

**DOI:** 10.1155/2021/6757916

**Published:** 2021-08-27

**Authors:** Sewnet Adem Kebede, Biruk Shalmeno Tusa, Adisu Birhanu Weldesenbet, Zemenu Tadesse Tessema, Tadesse Awoke Ayele

**Affiliations:** ^1^Department of Epidemiology and Biostatistics, Institute of Public Health, College of Medicine and Health Sciences, University of Gondar, Gondar, Ethiopia; ^2^Department of Epidemiology and Biostatistics, Collage of Health and Medical Sciences, Haramaya University, Haramaya, Ethiopia

## Abstract

**Background:**

Although the rate of diabetic nephropathy which is the leading cause of end-stage renal disease (ESRD) continues to rise, there is limited information about the problem. This study aimed to assess the incidence and predictors of diabetic nephropathy among type 2 DM patients.

**Methods:**

Institution-based retrospective follow-up study was conducted at UGCSH with 462 newly diagnosed type 2 DM patients from January 2001 to February 2016, and the data were collected by reviewing their records. The Schoenfeld residuals test was used to check proportional hazard assumption. The best model was selected by using Akaike information criteria (AIC). Hazard ratios (HR) with its respective 95% confidence interval were reported to show significance and strength of association.

**Results:**

The incidence rate of diabetic nephropathy was 14 (95% CI 10.8–17.7) cases per 10,000 patient-month observation. In addition, 63 (13.6%) DM patients developed diabetic nephropathy. The median time to develop diabetic nephropathy was 94.9 months with interquartile range (IOR) of (64.1–127.4) months. Type 2 DM patients who had coronary heart disease (AHR = 2.69, 95% CI 1.42–5.13) and anemia (AHR = 1.94, 95% CI 0.97–3.87) were at higher hazard for developing diabetic nephropathy. Besides this, having a long duration (>10 years) (AHR = 0.24, 95% CI 0.11–0.56) and being female (AHR = 0.44, 95% CI 0.26–0.73) was found to be protective against diabetic nephropathy.

**Conclusion:**

The incidence of diabetic nephropathy among type 2 diabetes patients remains a significant public health problem. Duration of diabetes >10 years and female sex reduced the risk of diabetic nephropathy. Coronary heart disease and anemia increased the risk of diabetic nephropathy among type 2 DM patients. In light of these findings, early screening for diabetes complication is needed, and health professionals should give targeted intervention for type 2 DM patients with coronary heart disease comorbidity and anemia.

## 1. Introduction

Diabetic nephropathy (DN) is one of the most common microvascular complications of diabetes and a leading cause of morbidity and mortality in diabetic patients [1, 2]. This condition is a result of vascular abnormalities that accompany diabetes and increases mortality risk [3]. It is also the leading cause of end-stage renal disease (ESRD) worldwide and a leading cause of DM-related morbidity and mortality [4, 5]. The proportion of ESRD attributable to diabetes alone ranges from 12% to 55% [1].

The rise in DN prevalence corresponds to the dramatic rise in diabetes prevalence around the world. Approximately 463 million adults aged 20–79 years are currently living with diabetes. Almost half (46.2%) of deaths associated with diabetes occur in people under the age of 60 years [6]. Due to the effect of globalization and epidemiologic transition, it is estimated that 79.4% of adults with diabetes live in low- and middle-income countries [7, 8]. In the United Kingdom, 25% of people with diabetes and in the United States of America, 36% of people with diabetes have diabetic nephropathy [6]. The prevalence of DN in Africa varied from 11% to 83.7% [9].

The pathogenesis of DN is complex and multifactorial. According to different literatures, the most common risk factors for development of diabetic nephropathy complication among type 2 diabetic patients include sex, age, body mass index, hypertension, duration, and fasting blood sugar [10–12]. Studies also have illustrated a tight relationship between diabetic nephropathy and diabetic retinopathy [13–15]. However, the importance of the above factors varies between studies.

The increasing prevalence of the diabetes mellitus is linked with the emergence of diabetes complication as a cause of premature death and disability. It is also associated with a negative economic impact for many countries [6, 16, 17]. People with diabetes and clinical nephropathy experience 50% higher health expenditures compared to those with diabetes but without clinical nephropathy [18]. Sustainable development goal three of the United Nations has targeted to reduce diabetes and its severity among other noncommunicable diseases such as cardiovascular diseases, cancers, and chronic respiratory diseases [19].

Several studies in Ethiopia have shown that the presence and severity of complications related to diabetic nephropathy like ESRD are steadily increasing, and these are the causes of premature death, disability, and negative economic impact [6, 16–18]. In developed countries, the incidence and risk factors of diabetic nephropathy have been well documented. However, studies regarding the incidence of diabetic nephropathy and its predictors are scarce in Ethiopia. To present, most of epidemiological research on diabetic nephropathy in Africa including Ethiopia has been limited to prevalence estimation from cross-sectional studies. Estimating the incidence of DN and early detection of the risk factors is important for the prevention of DN. Thus, we determined the incidence and predictors of diabetic nephropathy among type 2 diabetes patients.

## 2. Materials and Methods

### 2.1. Study Area

The study was conducted at University of Gondar Comprehensive Specialized Hospital, which is a teaching hospital. The hospital serves for greater than 5 million people in northwest Ethiopia. Around 24,862 numbers of people are having chronic follow-up per year, and among this, 8,900 are DM patients.

### 2.2. Study Design and Subjects

An institutional-based retrospective follow-up study was conducted. All newly diagnosed type 2 diabetes mellitus (T2DM) patients who are enrolled from January 2001 to February 2016 at University of Gondar Comprehensive Specialized Hospital were considered in this study. New T2DM diagnosed patients were eligible, while those who had diabetic nephropathy at the time of the diagnosis for T2DM were excluded from the study.

### 2.3. Sample Size and Sampling Technique

The required sample size was calculated via Stata software using power analysis for the log rank test by considering the following assumptions: survival probability of those having HDL <40 mg/dl (*p*=0.77) [10], since the HDL level is an independent risk factor for the development of microvascular disease affecting the kidney in patients with type 2 diabetes [20], 95% confidence level, and 5% margin of error. Therefore, the total calculated sample size was 462.

### 2.4. Data Collection Methods

The study used secondary data; a data extraction check list was prepared to collect the data. The reviewed records were identified by their medical registration number. Patient intake form follow-up card and DM registration book are used as data sources. Sociodemographic characteristics, baseline, and follow-up clinical and laboratory data were collected from patient cards. The date that patients start regular follow-up treatment until the end of the study to the confirmation of a final event in the study period was collected.

### 2.5. Data Quality Control Methods

A week before the actual data collection, preliminary review was done on similar area. The data extraction sheet was the pretest for consistency of understanding, adequacy of instrument, time requirement to fill the check list of tools, and completeness of data for charts. Necessary adjustment for the final data collection sheet was made by excluding variables which are not found on charts. Training on the objective of the study and how to retrieve records as per data extraction sheet was given to data collectors and supervisors two days before data collection. As well as, random sample from data extracted was crosschecked for its consistency. The information formats were crosschecked with the source card on the spot, and regular supervision was done.

### 2.6. Data Processing and Analysis

After they were checked for completeness, data were entered using Epi Info 7 and exported to STATA 14 for further analysis. The outcome variable in this study was time to diabetic nephropathy. Diabetic nephropathy was defined as an estimated glomerular filtration rate (eGFR) < 60 ml/min/1.73 m^2^ estimated by the Cockcroft–Gault equation. [21, 22] Accordingly, participants were classified as either diabetic nephropathy cases or censored at the end of the study. Furthermore, the incidence of diabetic nephropathy was determined from the start of type 2 DM diagnosis until the last follow-up visit.

We used the Weibull regression model to identify the predictors of diabetic nephropathy. Person-time at risk was measured starting from the time of initiation of treatment until each patient ended the follow-up. The Schoenfeld residuals test (both global and scaled) and graphical methods were used to check the Cox proportional hazard (PH) assumption. Cox PH and three parametric models (exponential, Weibull, and log-logistic) were fitted to identify the predictors of diabetic nephropathy. The best model was selected by using Akaike information criteria (AIC), Bayesian information criteria (BIC), and log likelihood criteria.

Goodness of fit of the model was assessed by using the Cox–Snell residual technique. Variables with a *p* value of ≤0.2 were entered into a multivariable model to control the possible effect of confounders. Variables having *p* value less than 0.05 in the multivariable model were considered significantly associated with the dependent variable. Hazard ratios (HR) with its 95% confidence interval were computed to determine statistical significance.

### 2.7. Ethical Consideration

Before the commencement of the study, ethical clearance was obtained from the Institutional Review Board of the University of Gondar. Then, permission letters from officials of University of Gondar Comprehensive Specialized Hospital, Department of Internal Medicine, were processed before data collection. To ensure confidentiality, patient names were not included; instead, code numbers were assigned to depict the results.

## 3. Results

### 3.1. Baseline Characteristics of Study Participants

Out of the total of 462 newly diagnosed type 2 DM patients, 277 (60%) were females. The mean duration of diabetes was 8.2 years (SD = ±3.8). More than half, 384 (82.9%) had no history of diabetic retinopathy. About 59 (12.8%) were on insulin. The mean (±SD) age for patients at the start of treatment was 53.2 (±10.1) years (Table 1).

### 3.2. Incidence of Diabetic Nephropathy

During the follow up, a total of 63 patients developed diabetic nephropathy. The median time to develop diabetic nephropathy was 94.9 months with an interquartile range (IOR) of 64.1–127.4 months. The overall incidence rate of diabetic nephropathy was 14 (95% CI 10.8–17.7) cases per 10,000 patient-month with total 45437.1 patient-month observation. Moreover, the proportion of diabetic nephropathy among newly diagnosed T2DM patients was 13.6%.

**Figure 1:**
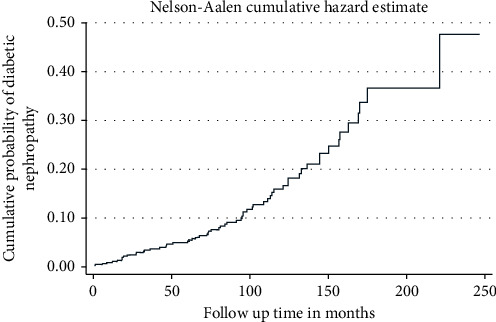
The Nelson–Aalen estimated cumulative curve showing cumulative probability of diabetic nephropathy among type 2 DM patients in University of Gondar Comprehensive Specialized Hospital, January 2001–February 2016.

The cumulative probability of developing diabetic nephropathy among type 2 DM patients who were free from diabetic nephropathy at the start of treatment was 0.0359 at month 40, 0.1117 at month 100, 0.3046 at month 180, and 0.3973 at month 230 during the follow-up period (Figure 1).

**Figure 2:**
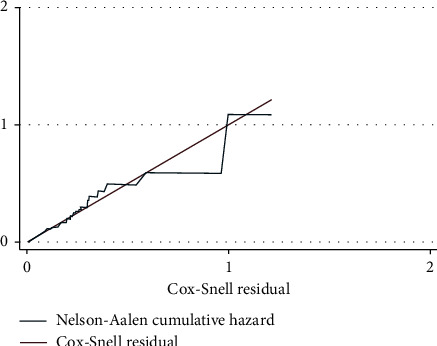
Cox–Snell residuals for Weibull PH models of newly diagnosed T2DM patients in University of Gondar Comprehensive Specialized Hospital, January 2001–February 2016.

Based on AIC, the Weibull–Cox regression model was the most efficient model to describe the data (AIC = 416.4). According to the Schoenfeld residual global test, the overall full model satisfies the proportional hazard assumption (*X*2 = 4.67, *p* < 0.912). As well, the Cox–Snell residual plot showed the proportional hazard assumption was satisfied (Figure 2).

### 3.3. Predictors of Diabetic Nephropathy among Type 2 DM Patients

Multivariable analysis result from fitted Weibull regression showed that sex, duration, systolic blood pressure, anemia, and coronary heart disease (CHD) were independent predictors for diabetic nephropathy among type 2 DM patients (Table 2).

The risk of developing diabetic nephropathy for patients who have CHD was 2.69 times higher than that of patients who have no CHD (AHR = 2.69, 95% CI 1.42–5.13). The risk of developing diabetic nephropathy was increased by 94% (AHR = 1.94, 95% CI 0.97–3.87) among newly diagnosed T2DM with anemia than patients with no anemia. The hazard of developing diabetic nephropathy was decreased by 76% among newly diagnosed type 2 diabetic patients with duration greater than 10 years than patients with duration less than 6 years (AHR = 0.24, 95% CI 0.11–0.56). The risk of developing diabetic nephropathy was decreased by 57% among female type 2 DM patients than male patients (AHR = 0.44, 95% CI 0.26–0.73).

## 4. Discussion

This study examined the incidence and predictors of diabetic nephropathy among newly diagnosed T2DM patients at University of Gondar Comprehensive Specialized Hospital. Duration of diabetes, CHD, anemia, and sex were found to be independent predictors of diabetic nephropathy.

The study revealed the incidence rate of diabetic nephropathy was 14 (95% CI 10.8–17.7) cases per 10,000 patient-months of observation. The proportion of diabetic nephropathy was 13.6% with 14 cases per 10,000 patient-month observation. The study showed a lower incidence of diabetic nephropathy than studies performed in Ethiopia and United Kingdom (UK) [10, 23]. This inconsistency might be due to the difference in the sample size, diagnostic method, and study design among the studies.

In this study, the median survival time was 94.9 months which is longer than the findings of previous studies performed in Ethiopia, 70.9 months, [10] and Honk Kong, 55.2 months [24]. This could be explained by the increase in access and variety of more potent drugs nowadays than the past periods.

The study identified that among type 2 DM patient's, women had a longer time to develop diabetic nephropathy than men. This result is in line with the studies performed in France [25] and Sweden [26]. This may be explained by the fact that the estrogen hormone plays an important role in protection [27]. The other possible explanation could be that renal function in women is underestimated if creatinine-based estimates of the glomerular filtration rate are used despite gender adjustments. In contrast to our results, other studies [23, 24, 28–31] showed that men had a lower risk of developing microvascular complications of DM than women. Therefore, further research is needed to determine whether this gender difference contributes to better outcomes in females with diabetes.

The current study showed that the duration of diabetes was negatively associated with hazard of diabetic nephropathy. This finding is inconsistent with previous studies [24, 27, 32–34]. This might be since our study populations were type 2 DM patients who are more likely to come late to the health facility and seek health services because the disease process of T2DM is more gradual and not as severe as type 1 DM at the early stage of the disease. According to American Diabetes Association medical care standards, diabetes kidney disease may be present at the diagnosis of type 2 diabetes [2]. That is why duration of diabetes and diabetic nephropathy is inversely related in our study. Since the association between duration and diabetic nephropathy is controversial, it needs further research.

We found that CHD is a risk factor for diabetic nephropathy. The finding is in agreement with a previous study [35] which shows that the cell adhesion molecules are raised in both cardiovascular disease and diabetic nephropathy, with levels increasing in stepwise fashion with increasing kidney disease. The possible justification could be that coronary artery stenosis increased renal oxidative stress, fibrosis, inflammation, tubular injury, and microvasculature remodelling [36]. This finding is inconsistent with the study performed in Spain [37]. This is unclear and needs further investigation.

This study found that the presence of anemia was an independent risk factor for diabetic nephropathy. This finding is in line with previous studies performed in USA [11], China [38], and Kenya [39]. This could be linked to chronic hyperglycemia which is involved in the pathogenesis of anemia by causing abnormalities in red blood cells, oxidative stress, autonomic neuropathy, and renal sympathetic denervation. These conditions put the renal interstitium in a hypoxic state, and consequently, the production of erythropoietin by peritubular fibroblasts is impaired.

The strength of this study was following patients for long duration. This study is not free from limitation. Since the data were secondary data, some potentially important predictors were not available like smoking behavior, family history of diabetes, and kidney disease. Furthermore, use of secondary data collected retrospectively results in incompleteness.

## 5. Conclusion

In this retrospective follow-up study, findings showed that the incidence of diabetic nephropathy among type 2 diabetes mellitus patients remains a significant public health problem. Duration of diabetes >10 years and female sex reduced the risk of diabetic nephropathy. Besides this, coronary heart disease and anemia increased the risk diabetic nephropathy among type 2 diabetes mellitus patients. In light of these findings, health professionals in the DM follow-up clinics should give targeted intervention for type 2 DM patients with coronary heart disease comorbidity and anemia. Early screening and treatment of diabetes complications would be an essential part of DM care, delaying the onset of diabetic nephropathy.

## Figures and Tables

**Table 1:** Sociodemographic and clinical factors of newly diagnosed T2DM patients in University of Gondar Comprehensive Specialized Hospital, January 2001–February 2016.

Variable	Censored (%)	Diabetic nephropathy (%)	Total
Sex			
Male	149 (37.3)	36 (57.1)	185 (40.0)
Female	250 (62.7)	27 (42.9)	277 (60.0)

Age	Mean = 53.2 ± 10.1		
≤59	278 (69.7)	43 (68.3)	321 (69.5)
≥60	121 (30.3)	20 (31.7)	141 (30.5)

Diabetic retinopathy			
Yes	68 (17.0)	11 (17.5)	79 (17.1)
No	331 (83.0)	52 (82.5)	383 (82.9)

Diabetic neuropathy			
Yes	65 (16.3)	13 (20.6)	78 (16.9)
No	334 (83.7)	50 (79.4)	384 (83.1)

Hypertension			
Yes	89 (22.3)	19 (30.2)	108 (23.4)
No	310 (77.7)	44 (69.8)	354 (76.6)

Anemia			
Yes	26 (6.5)	12 (19.1)	38 (8.2)
No	373 (93.5)	51 (80.9)	424 (91.8)

Medication			
Dietary modification	21 (5.3)	8 (12.7)	29 (6.3)
One oral agent	261 (65.4)	34 (54.0)	295 (63.8)
>1 oral agent	70 (17.5)	9 (14.3)	79 (17.1)
Insulin	47 (11.8)	12 (19.1)	59 (12.8)

Duration (year)	Mean = 8.2 ± 3.8		
<6	100 (25.1)	11 (17.5)	111 (24.0)
6–10	199 (49.8)	32 (50.8)	231 (50.0)
>10	100 (25.1)	20 (31.7)	120 (26.0)

FBS	Mean = 224.6 ± 80.9		
≤150	83 (20.8)	8 (12.7)	91 (19.7)
>150	316 (79.2)	55 (87.3)	371 (80.3)

FBS, fasting blood sugar.

**Table 2:** Multivariable analysis using the Weibull regression model for predictors of diabetic nephropathy among type 2 DM patients in University of Gondar Comprehensive Specialized Hospital, January 2001–February 2016.

Variable	Crude HR (95% CI)	Adjusted HR (95% CI)
Sex		
Male	1	1
Female	0.44 (0.27–0.73)	0.44 (0.26–0.73)^*∗∗∗*^

FBS		
≤150	1	1
>150	1.41 (0.67–2.96)	1.59 (0.75–3.39)

Duration		
<6 years	1	1
6–10 years	0.53 (0.26–1.06)	0.50 (0.24–1.04)
>10 years	0.28 (0.13–0.62)	0.24 (0.11–0.56)^*∗∗∗*^

SBP		
≤140	1	1
>140	1.31 (0.75–2.29)	1.91 (0.96–3.67)

DBP		
≤90	1	1
>90	0.67 (0.29–1.57)	0.53 (0.19–1.41)

Anemia		
No	1	1
Yes	2.93 (1.56–5.51)	1.94 (0.97–3.87)^*∗*^

Diabetic retinopathy		
No	1	1
Yes	0.78 (0.41–1.50)	0.71 (0.36–1.41)
Age (year)	1.02 (0.99–1.04)	1.01 (0.98–1.03)

CHD		
No	1	1
Yes	2.91 (1.61–5.27)	2.69 (1.42–5.13)^*∗∗*^

^*∗∗∗*^*P* value < 0.001, ^*∗∗*^*p* value < 0.01, ^*∗*^*p* value < 0.05. CI, confidence interval; DBP, diastolic blood pressure; FBS, fasting blood sugar; SBP, systolic blood pressure; CHD, coronary heart disease.

## Data Availability

The data used to support the findings of this study are included within the article.

## Abbreviations

AHR: Adjusted hazard ratio
AIC: Akaike's information criterion
BIC: Bayesian information criterion
CHD: Coronary heart disease
CHR: Crude hazard ratio
DM: Diabetes mellitus
ESRD: End-stage renal disease
HR: Hazard ratio
PH: Proportional hazard
T2DM: Type 2 diabetes mellitus.
